# Factors Associated With New Analgesic Requirements Following Critical Illness

**DOI:** 10.1177/08850666231219916

**Published:** 2023-12-12

**Authors:** Mark Andonovic, Martin Shaw, Tara Quasim, Pamela MacTavish, Joanne McPeake

**Affiliations:** 1 Academic Unit of Anaesthesia, Critical Care and Perioperative Medicine, 3526 University of Glasgow, Glasgow, UK; 2 Department of Pharmacy, 59736 Glasgow Royal Infirmary, Glasgow, UK; 3 The Healthcare Improvement Studies Institute, 2152 University of Cambridge, Cambridge, UK; 4 1016 Healthcare Improvement Scotland, Edinburgh, UK

**Keywords:** analgesics, opioids, pain, critical care, long-term outcomes

## Abstract

**Background:**

Chronic opioid use represents a significant burden to global healthcare with adverse long-term outcomes. Elevated patient reported pain levels and analgesic prescriptions have been reported following discharge from critical care. We describe analgesic requirements following discharge from hospital and identify if a critical care admission is a significant factor for stronger analgesic prescriptions.

**Methods:**

This retrospective observational cohort study identified patients in the UK Biobank with a registered admission to any UK hospital between January 1, 2010 and December 31, 2015 and information on prescriptions drawn both prior to and following hospital discharge. Two matched cohorts were created from the dataset: critical care patients and hospital patients admitted without a critical care encounter. Outcomes were analgesic requirements following hospital discharge and factors associated with increased analgesic prescriptions. Multivariable logistic regression was used to identify factors associated with prescriptions from higher steps on the World Health Organization (WHO) analgesic ladder.

**Results:**

In total, 660 formed the total study population. Strong opioid prescriptions following discharge were significantly higher in the critical care cohort (*P* value <.001). Critical care admission (OR = 1.45) and increasing Townsend deprivation (OR = 1.04) index were significantly associated with increasing strength of analgesic prescriptions following discharge.

**Conclusions:**

Critical care patients require stronger analgesic prescriptions in the 12 months following hospital discharge. Patients from areas of high socioeconomic deprivation may also be associated with increased analgesic requirements. Multidisciplinary support is required for patients who may be at risk of chronic opioid use and could be delivered within critical care recovery programs.

## Background

Chronic opioid use represents a significant burden to global healthcare and commonly results in population morbidity and mortality, with recent figures suggesting that an estimated 40.5 million people are dependent worldwide.^1^ In addition to this, the rates of opioid-related deaths doubled in certain areas of the UK over a subsequent 10-year period.^2^ The National Institute on Drug Abuse reported 80 411 overdose deaths involving any opioids across the USA in 2021: this was almost 4 times greater than the reported number of opioid-related deaths in 2010.^3^ Iatrogenic prescribing has been recognized as an important cause of chronic opioid use, with a recent meta-analysis estimating a pooled incidence of opioid dependence or abuse of 4.7% among patients prescribed opioids.^4^

The factors that have been described as contributing to long-term prescribing and subsequent chronic use included acute emergency admission to hospital and the perioperative period for both emergency and elective patients requiring surgery.^5^ Recent data has also demonstrated that critically unwell patients have a higher risk of discharge on opioid prescriptions regardless of whether they are prescribed opioids prior to admission.^6,7^ This may be attributable to the opioid infusions which critically unwell patients frequently receive while on invasive mechanical ventilation as part of the requirement for analgesia during their critical illness^8^; a survey conducted across adult general critical care units demonstrated that 82.7% of units used a combination of a sedative agent and an opioid as the standard practice for sedation.^9^

In conjunction with the above, a significant body of literature has established that admission to intensive care can impact patients long after discharge from hospital, with the recognition that many patients will go on to develop long-term problems, including the development of chronic pain.^10,11^ For example, a recent cohort study of Intensive Care Unit (ICU) patients demonstrated that almost 60% of patients described pain not in keeping with everyday pain 1 year following discharge^12^; a further study which followed up ICU survivors for 1 year after discharge found that 66% of patients reported a new chronic pain that did not exist before their stay in the ICU.^13^ Given the high prevalence of ongoing pain in these patients long after discharge, coupled with often protracted opioid infusions during their inpatient stay, it is understandable why these patients are at higher risk for subsequent chronic opioid use regardless of opioid use prior to admission.^14^ Given this higher risk, patients admitted to critical care may have higher analgesic requirements than patients admitted to the general hospital.

We aimed to understand opioid use following critical illness and subsequently identify potential factors which may contribute to increased analgesic requirements. Furthermore, we identified previously “opioid naïve” patients to understand opioid use in these patients following critical illness when compared with hospitalized patients not admitted to critical care. We hypothesized that critical care survivors would have an increased requirement for analgesia following discharge from hospital compared with a general inpatient cohort.

## Methods

### Design

This retrospective observational cohort study utilized data which were collected and via the UK Biobank repository. The UK Biobank study was approved by the Northwest Multicentre Ethics Research Committee; the participants provided written informed consent and agreed to have their health followed longitudinally, via linkage to routine clinical data (including healthcare resource use and diagnostic data). This study is part of the UK Biobank project 57617 (NHS National Research Ethics Service No. 11/NW/0382). Data for this analysis was censored at December 31, 2016 to ensure that a 12-month follow-up data was available for all patients included in the study. All patients who have withdrawn consent from the UK Biobank were removed from this analysis. Data in the UK Biobank are linked to routinely held NHS data annually. The findings are reported based on Strengthening the Reporting of Observational Studies in Epidemiology (STROBE) guidelines.^15^

### Setting and Participants

This study was conducted utilizing data from the UK Biobank. This database houses data from over 500 000 volunteers from across the UK and links records from primary and secondary care to build a comprehensive health record on each patient. Patients eligible for inclusion in the study must have had a registered hospital admission to any UK hospital between January 1, 2010 and December 31, 2015 and survived to hospital discharge. Patients also required information on prescriptions drawn from primary care both prior to admission and following hospital discharge. The full UK Biobank was prescreened for any patient with at least 1 prescription from chapter 4 section 7 of the British National Formulary (BNF) which contains all analgesic medications,^16^ and this dataset was used for initial screening for eligibility. Patients were excluded if they were less than 18 years old or if they were prescribed Methadone in the community—this drug is predominantly used as part of treatment for opioid use disorder within the UK.

### Definition of Variables

Patients included in the patient population were dichotomized based on if they were admitted to a critical care for at least 1 day at any point during their admission; this data was available as a data field within the dataset and was used to form the 2 cohorts of critical care patients and hospital patients. Critical care admission was defined in this cohort by the Consultant speciality available in the UK Biobank. Patients admitted to critical care were matched with a cohort of general hospitalized inpatients: 1:1 propensity score matching using k-nearest neighbors was conducted based on matches of age, sex, Townsend deprivation index, total hospital length of stay, type of admission, and ethnic background. This produced matched cohorts with similar demographics and factors related to their acute illness which may contribute to long-term opioid use; these cohorts were therefore more likely to delineate if admission to critical care was independently associated with increased analgesic requirements.

The Townsend index is used a measure of deprivation in specific areas or patients^17^: this utilizes a census-based index of material deprivation calculated by combining 4 variables with positive values indicating higher material deprivation and a score of 0 representing an area with overall mean values. The Townsend index is calculated automatically for each patient added to the UK Biobank dataset. Patient admissions were also categorized as elective (planned) or emergency (unplanned). Finally, each patient admission was defined in the Biobank as being under a medical specialty or a surgical specialty.

For subgroup analyses, prescription data prior to hospital admission was used to define an “opioid naïve” population. Patients were defined as opioid naïve if they had not drawn an opioid prescription in the 12 months prior to their admission date. To allow for comparison of analgesic requirements before and after inpatient hospital stay, patient prescriptions were graded according to the World Health Organization (WHO) analgesic ladder.^18^ The maximum grade of WHO ladder analgesic was defined as the highest level of filled prescription at any time point in the 12 months before admission (Max WHO ladder before) or in the 12 months after discharge (Max WHO ladder after); this methodology has previously been used to understand analgesic requirements in the context of critical illness.^19,20^ Patients with at least 1 prescription filled within 2 consecutive 3-month periods were classified as chronic opioid users: this included opiates on either stage 2 or stage 3 on the WHO analgesic ladder. As it is commonly used for secondary prevention following major cardiovascular events, Aspirin was not included as an analgesic in these analyses.

### Statistical Analyses

All statistical analyses were conducted using the software package R (The R Foundation; R version 4.2.2). Variables were summarized using median values and interquartile range (IQR) or by proportions with 95% confidence interval (95%CI); difference in median values were compared using the Wilcoxon rank-sum test, whereas difference in proportions were compared using the Pearson Chi-squared test. The propensity score matching process was also conducted using the “MatchIt” package in R: it utilized 1:1 matching using k-nearest neighbor without the use of calipers. Regression analysis was used to identify factors associated with increased levels on the WHO analgesic ladder. Initial univariable analyses were performed on each collected variable. A directed acyclic graph (DAG) was then constructed to determine the relationship of collected variables compared to the exposure and the outcome: if a variable was found to be a confounder, it was selected for inclusion in the multivariable model. An exploratory multivariable logistic regression model was then used to calculate adjusted odds ratios (OR) and 95% CIs. For all the analyses, a statistical significance was set at a 2-sided *P* value of <.05. Patients with chronic opioid use prior to admission were initially examined as a factor for stronger prescriptions following discharge; however, this effect was found to be so strong on multivariable analysis that it masked the effect of other variables. A sensitivity analysis was conducted, and it found that factors significantly associated with chronic opioid use prior to admission were similar to the final results; therefore, it was considered very closely related and reasonable to omit from the final adjusted analysis.

### Outcomes

The primary outcomes for this study were analgesic requirements following discharge in patients admitted to critical care compared with the general hospital population. Secondary outcomes included identification of factors which may be associated with increased analgesic requirements and differences found when analyzing a subgroup of opioid naïve patients.

## Results

**Figure 1.**
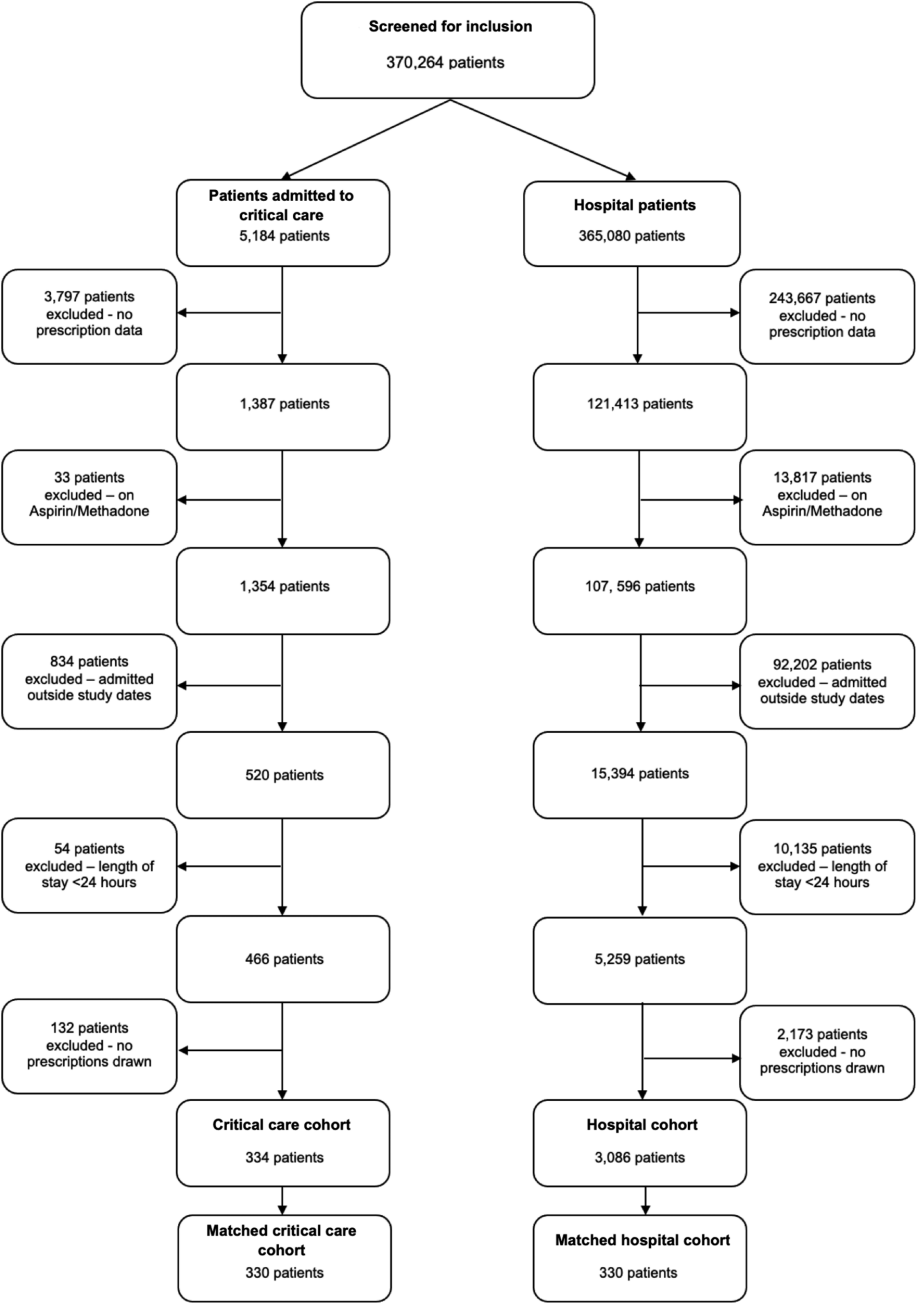
Flow diagram of patients.

The 370 264 patients with at least 1 analgesic prescription were initially screened for eligibility: of these, 334 patients were admitted to critical care during their inpatient admission and met all the eligibility criteria. The matching of these patients based on our predefined matching criteria identified 330 patients who were matched with the hospital cohort; an exact match could not be identified for 4 patients in the critical care cohort. These patients and their matched critical care patients formed the final study population of 660 patients (Figure 1). No missing data were identified in this study population which included a total follow-up period of 12 months for all patients.

### Baseline Characteristics

Baseline demographics for the study population can be found in Table 1. The median age of the critical care population was found to be significantly higher than the hospital population (67.0 vs 66.0, respectively; *P* value = .011); the proportion of elective admissions, type of admission, patient sex, Townsend deprivation index, ethnic background, and hospital length of stay were similar between the 2 groups (Table 1).

**Table 1.** Characteristics of the Total Study Population Based on Admission to Critical Care.

	Overall (*n* = 660)	Hospital cohort (*n* = 330)	Critical care cohort (*n* = 330)	*P* value
Age—median (IQR)	66 (61-70)	66 (60-69)	67 (62-70)	0.011
Male sex—*n* (%)	389 (58.9%)	190 (57.6%)	199 (60.3%)	0.500
Surgical admission—*n* (%)	605 (91.7%)	299 (90.6%)	306 (92.7%)	0.300
Emergency admission—*n* (%)	471 (71.4%)	245 (74.2%)	226 (68.5%)	0.100
Townsend deprivation index—median (IQR)	−1.6 (−3.4–1.7)	−1.8 (−3.5–1.5)	−1.4(−3.3–1.8)	0.200
Ethnic background				0.800
White	617 (93.5%)	309 (93.6%)	308 (93.3%)	
Black	5 (0.8%)	3 (0.9%)	2 (0.6%)	
South Asian	22 (3.3%)	9 (2.7%)	13 (3.9%)	
Other	16 (2.4%)	9 (2.7%)	7 (2.1%)	
Critical care length of stay—median (IQR)	0 (0-3)	0 (0-0)	3 (1-6)	<0.001
Hospital length of stay—median (IQR)	13 (6-28)	14 (6-25)	13 (6-33)	0.300

### Analgesic Requirements Following Discharge

The number of analgesic prescriptions filled in the 12 months following hospital discharge were identified, and the median values for critical care patients and hospital patients were calculated: no significant between-group differences were seen (Table 2). Similarly, no significant between-group difference was seen in chronic opioid use following over the total 12-month period following hospital discharge (*P* value = .700). However, a significantly higher proportion of patients in the critical care cohort were found to have drawn a strong opioid prescription following discharge from hospital when compared to the hospital cohort (21.2% vs 14.5%; *P* value = .030).

**Table 2.** Outcomes of the Total Study Population Based on Admission to Critical Care.

	Overall (*n* = 660)	Hospital cohort (*n* = 330)	Critical care cohort (*n* = 330)	*P* value
Prescription count after discharge—median (IQR)	3 (1-8)	3 (1-7)	2 (0-10)	0.700
Max WHO analgesic ladder after discharge—*n*(%)				<0.001
None	140 (21.2%)	51 (15.5%)	89 (27.0%)	
Nonopioid (step 1)	175 (26.5%)	104 (31.5%)	71 (21.5%)	
Weak opioid (step 2)	227 (34.4%)	127 (38.5%)	100 (30.3%)	
Strong opioid (step 3)	118 (17.9%)	48 (14.5%)	70 (21.2%)	
Chronic opioid prescription after discharge—*n* (%)	246 (37.3%)	125 (37.9%)	121 (36.7%)	0.700

### Increased Strength of Analgesic Prescriptions

The initial univariable analyses of factors which may be associated with at least 1 increase in step on the WHO analgesic ladder can be found in Table 3: for the purpose of these analyses, an increase was defined as patients who had a prescription from a higher step on the analgesic ladder after discharge than they did prior to admission. Admission to critical care was found to be significantly associated with an increase in potency of prescribed analgesia across all steps of the analgesic ladder in the 12 months following hospital discharge (OR = 1.38; *P* value = .030). The only other factor to be significantly associated with a difference in analgesic requirement in the entire study population was increasing Townsend deprivation index: an increase of 1 in the Townsend index was associated with an odds ratio of 1.04 (*P* value <.001), which suggested an association between increased deprivation and increased opioid usage. The directed acyclic graph used to determine inclusion in the multivariable model can be found in Supplementary Figure 1. On exploratory multivariable analysis, both admission to critical care and increasing Townsend index remained significantly associated with an increased step in WHO analgesic requirement (OR = 1.45 and OR = 1.04, respectively). In addition, emergency admission was found to be significantly associated with reduced odds of requiring higher levels of analgesia (OR = 0.83; *P* value = .035).

**Table 3.** Stronger Analgesic Prescriptions Following Hospital Discharge.

	Univariable odds ratio (95% C.I.)	*P* value	Multivariable odds ratio (95% C.I.)	*P* value
Admission to critical care	1.38 (1.03-1.84)	0.030	1.45 (1.07-1.94)	0.019
Age	0.99 (0.98-1.01)	0.303	0.99 (0.98-1.01)	0.309
Male sex	0.91 (0.75-1.11)	0.343	0.87 (0.70-1.05)	0.162
Emergency admission	0.85 (0.70-1.03)	0.098	0.83 (0.68-0.99)	0.035
Townsend deprivation index	1.04 (1.01-1.07)	0.020	1.04 (1.01-1.07)	0.026
Surgical admission	1.09 (0.87-1.37)	0.455	1.07 (0.84-1.36)	0.580
Ethnic background			—	—
White	Ref	—		
Black	1.04 (0.45-2.39)	0.930		
South Asian	1.13 (0.65-2.00)	0.660		
Other	1.63 (0.73-3.70)	0.235		
Hospital length of stay	1.00 (1.00-1.00)	0.478	—	—

### Opioid Naïve Patients

Of the 660 patients forming the total study population, 434 were found to be opiate naïve prior to admission: baseline demographics for this subgroup of patients can be found in Supplementary Table 1. The median age was similar in opiate naïve patients admitted to critical care and those admitted to the general hospital population. Similar proportions of elective admissions, type of admission, patient sex, Townsend deprivation index, and ethnic backgrounds were seen in the critical care cohort and the hospital cohort. However, the median length of hospital stay was significantly longer in patients admitted to critical care, with 13.0 days compared to 10.0 days in the hospital group (*P* value <.001).

The outcomes for the 434 opiate naïve patients can be found in Supplementary Table 2. The median number of analgesic prescriptions filled in the 6 months following hospital discharge was similar in both the critical care group and the general hospital group (3 and 4 respectively; *P* value = .500). Proportions of patients classified as using chronic opioids were also similar in both groups, with 96 patients out of 217 (44.2%) in the hospital group compared with 94 out of 217 (43.3%) in the hospital group (*P* value = .800). The patients in the hospital group were found to a have a significantly greater likelihood of filling a nonopioid analgesic prescription (32.7% vs 19.4%; *P* value <.001), while critical care patients were more likely to fill a prescription for a strong opioid at any point in the 12 months following discharge compared with hospital patients (23.5% vs 12.9%; *P* value <.001).

When analyzing factors associated with increasing analgesic requirements, initial univariable analyses were conducted on all available variables: these results can be found in Table 4. The only factor found to be statistically significant was the type of admission; these results demonstrated that emergency admission was associated with reduced odds of requiring higher levels of analgesia over the total 12-month period following discharge from hospital (OR = 0.55; *P* value <.001). On a subsequent multivariable analysis, this association was retained, with an adjusted odds ratio of 0.57 (*P* value <.001). While sex was not found to be statistically significant on initial univariable analysis, a multivariable analysis revealed that male sex was associated with reduced odds of drawing prescriptions from higher steps on the WHO analgesic ladder after discharge from hospital (OR = 0.70; *P* value = .040). Age and admission to intensive care were not found to be significantly associated with increasing analgesic requirements following discharge on either univariable or multivariable analyses. Unlike with the total study population, deprivation was not found to be a significant factor in an increase in step on the WHO analgesic ladder.

**Table 4.** Stronger Analgesic Prescriptions Following Hospital Discharge in Opioid Naïve Patients.

	Univariable odds ratio (95% C.I.)	*P* value	Multivariable odds ratio (95% C.I.)	*P* value
Admission to critical care	1.17 (0.83-1.64)	0.373	1.15 (0.79-1.68)	0.515
Age	0.99 (0.96-1.01)	0.329	0.99 (0.97-1.01)	0.372
Male sex	0.71 (0.51-1.00)	0.053	0.70 (0.51-0.97)	0.040
Emergency admission	0.55 (0.38-0.78)	<0.001	0.57 (0.40-0.79)	<0.001
Townsend deprivation index	1.00 (0.95-1.05)	0.859	1.00 (0.94-1.05)	0.822
Surgical admission	1.29 (0.70-2.38)	0.412	1.20 (0.64-2.41)	0.599
Ethnic background			—	—
White	Ref	—		
Black	0.50 (0.09-2.62)	0.407		
South Asian	0.89 (0.41-1.89)	0.754		
Other	2.96 (0.55-17.00)	0.200		
Hospital length of stay	1.00 (0.99-1.00)	0.193	—	—

## Discussion

This study has demonstrated that in a matched cohort, derived from a large UK-based data repository, admission to critical care was significantly associated with increased analgesic requirements over the first 12 months following discharge from hospital. Furthermore, increased deprivation was significantly associated with prescriptions on a higher step on the WHO analgesic ladder following hospital discharge. In the analyses of both the main study population and a subgroup of opiate naïve patients, emergency admission to hospital was significantly associated with reduced odds of requiring medication from higher steps on the WHO analgesic ladder over the total 12-month follow-up period.

The association found in this study between admission to critical care and requiring stronger analgesic prescriptions may be explained by significantly higher rates of reported chronic pain following critical illness; Griffiths and colleagues^21^ conducted a UK-based study which found that 73% of ICU survivors reported chronic pain at 12 months. Given the heterogenous nature of these pain among ICU survivors, careful attention is required in the postdischarge period to support patients with pain management and control. Delivery of these interventions should ideally be both multifaceted and multidisciplinary, with input from clinicians, pharmacists, and clinical psychologists. As described by Connolly et al, ^22^ ICU recovery programs have become more common throughout the UK and frequently involve healthcare professionals from multiple disciplines.^22^ Such recovery programs may offer the ideal environment in which to address pain management and long-term analgesic prescriptions. Future research should specifically address how ICU follow-up services can contribute to helping manage these identified issues.

This study identified a potential association between increased socioeconomic deprivation and prescriptions for stronger analgesics following discharge from hospital. Prior population-based studies have demonstrated a well-established link between areas with high levels of deprivation and higher rates of opioid use and filled opioid prescriptions in both the UK and USA.^23,24^ In a comprehensive report compiled by the UK parliament, drug use disorders in Scotland were found to be 17 times more prevalent in the country's most deprived areas^25^: this has a significant degree of crossover with the association seen between deprivation and prescriptions drawn from higher steps on the WHO analgesic ladder which comprise of opioids. The hypothesized reasons behind this were multifactorial, citing additional risk factors such as unstable home life, unemployment, and substance misuse arising due to self-medication for chronic health issues.^25^ This report also went on to state that people from deprived areas were less likely to overcome these problems as they have less access to factors that support recovery. As an individualized DAG was not created for socioeconomic deprivation, causality cannot be inferred, but this demonstrates an important area for future analysis. This also further highlights that individuals need targeted support to fully elucidate their need for stronger analgesia following discharge and potentially identify methods to help address the problem and prevent it from becoming a gateway to long-term opioid use.

Emergency admissions were found to be potentially associated with reduced odds of requiring stronger analgesic prescriptions across the total study population and in the opioid naïve subgroup. This is at odds to prior data, which has previously stated that patients with elective admissions report lower pain levels and draw fewer analgesic prescriptions than elective admissions due to well-established and honed enhanced recovery after surgery (ERAS) pathways.^26,27^ The reason for this is unclear, but it may be explained by the fact that the matching process included total hospital length of stay, and therefore identified patients with more complex elective procedures requiring both longer hospital stays and increased analgesic requirements. The only other association identified during this study was that male patients were at reduced odds of requiring stronger analgesic prescriptions following hospital discharge. Previous studies have reported higher levels of postoperative pain and analgesic consumption in female patients,^28^ hypothesized to be due to differences in gonadal hormones which can modulate both pain intensity and influence sensitivity to opioid analgesics.^29^ This further serves to highlight the complex interplay of factors involved in increased analgesic requirements postdischarge and the benefit of an individualized process targeted to identify reasons behind stronger analgesic requirements and help to support and manage patients through the discharge and recovery process.

The large undifferentiated population included within the UK Biobank was a key strength of this study, and the prehospital and postdischarge data allowed for an in-depth analysis regarding analgesic requirements in a group of UK patients. However, this study has limitations. First, although comprehensive prescribing data was available for patients included in the study population, a large proportion of patients in the UK Biobank were lost due to incomplete prescription data, limiting the study size and generalizability relative to the whole UK Biobank cohort. In addition to this, the recruitment to the UK Biobank only represents 5.5% of the total invitations to participate, and therefore the characteristics of this group may not be representative of the total UK population. This self-selecting nature of the population may also result in significant selection bias. As the time frame for inclusion in this study is long, it may be that pain management at the beginning of the study differs from patients admitted toward the end of the study period. Second, a substantial proportion of UK Biobank patients are surgical, and therefore, despite adjustment for the type of admission in adjusted analyses, the results may not be as representative to patients admitted for medical reasons. Third, while we reported on the number of analgesic prescriptions following discharge, this does not consider the variable nature of duration over which the prescriptions are issued over the total 12-month follow-up period. We also only reported on maximum strength of prescription following discharge, and this may also have fluctuated in terms of analgesic requirements over the 12-month period. We were unable to include acute pain scores and inpatient use of opioids into the multivariable models due to the lack of inpatient data, which represents an important unadjusted confounder. Finally, the participants recruited to the UK Biobank are on average healthier and more likely to live in less socioeconomically deprived areas than the general UK population, which may limit the interpretability of these results.^30^

This study found that patients admitted to critical care are significantly associated with stronger levels of analgesic prescriptions in the 12 months following discharge. There was a potential association identified between stronger analgesic prescriptions and higher levels of socioeconomic deprivation which requires further study. Multidisciplinary support mechanisms could be implemented to support these patients through the hospital discharge periods, and this could ideally be delivered in the rapidly growing environment of critical care recovery programs.

## Supplemental Material

sj-docx-1-jic-10.1177_08850666231219916 - Supplemental material for Factors Associated With New Analgesic Requirements Following Critical Illness Supplemental material, sj-docx-1-jic-10.1177_08850666231219916 for Factors Associated With New Analgesic Requirements Following Critical Illness by Mark Andonovic, Martin Shaw, Tara Quasim, Pamela MacTavish and Joanne McPeake in Journal of Intensive Care Medicine

sj-docx-2-jic-10.1177_08850666231219916 - Supplemental material for Factors Associated With New Analgesic Requirements Following Critical Illness Supplemental material, sj-docx-2-jic-10.1177_08850666231219916 for Factors Associated With New Analgesic Requirements Following Critical Illness by Mark Andonovic, Martin Shaw, Tara Quasim, Pamela MacTavish and Joanne McPeake in Journal of Intensive Care Medicine
