# Supplementary material for: Factors Associated With New Analgesic Requirements Following Critical Illness
Source: J Intensive Care Med. 2023 Dec 12;39(6):550–7. doi: 10.1177/08850666231219916 (PMC11092297; doi:10.1177/08850666231219916)
Supplement: sj-docx-1-jic-10.1177_08850666231219916 - Supplemental material for Factors Associated With New Analgesic Requirements Following Critical Illness [file sj-docx-1-jic-10.1177_08850666231219916.docx]

**Supplementary Figure 1: Directed acyclic graph to determine association of variables and inclusion in multivariable models**

**
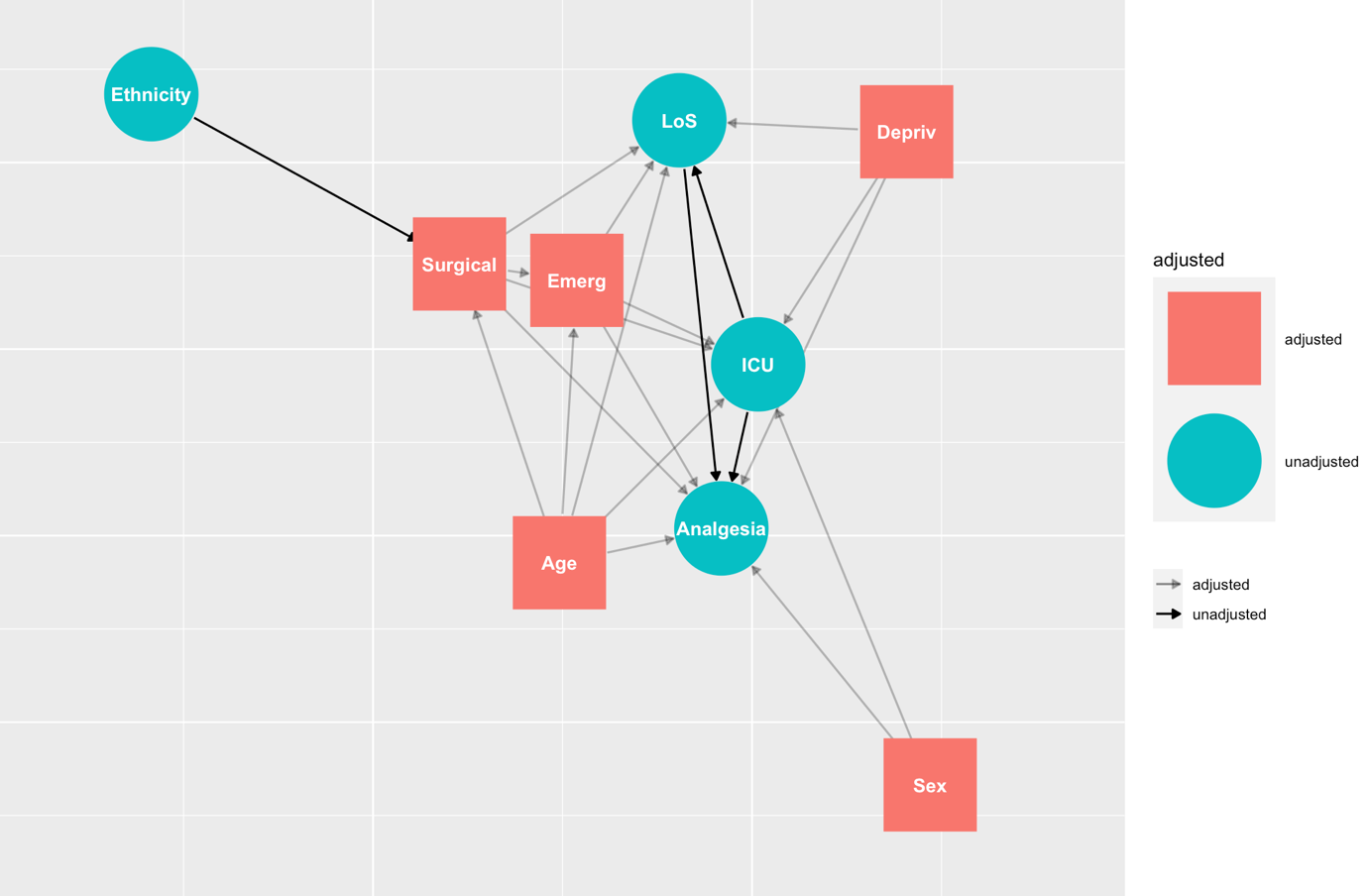
**

**Supplementary Table 1: Characteristics of opioid naïve patients based on admission to critical care**

|  | Overall  (n = 434) | Hospital cohort  (n = 217) | Critical care cohort  (n = 217) | *p*-value |
| --- | --- | --- | --- | --- |
| Age – median (IQR) | 67 (62 – 70) | 67 (62 – 70) | 67 (63 – 71) | 0.140 |
| Male sex – n (%) | 245 (56.5%) | 120 (55.3%) | 125 (57.6%) | 0.600 |
| Surgical admission – n (%) | 400 (92.2%) | 196 (90.3%) | 204 (94.0%) | 0.200 |
| Emergency admission – n (%) | 287 (66.1%) | 148 (68.2%) | 139 (64.1%) | 0.400 |
| Townsend deprivation index – median (IQR) | -1.6 (-3.3 – 1.9) | -1.8 (-3.4 – 2.1) | -1.2 (-3.3 – 1.6) | 0.400 |
| Ethnic background  White  Black  South Asian  Other | 406 (93.5%)  4 (0.9%)  19 (4.4%)  5 (1.2%) | 205 (94.5%)  2 (0.9%)  8 (3.7%)  2 (0.9%) | 201 (92.6%)  2 (0.9%)  11 (5.1%)  3 (1.4%) | 0.900 |
| Critical care length of stay – median (IQR) | 0 (0 – 2) | 0 (0 – 0) | 2 (1 – 5) | <0.001 |
| Hospital length of stay – median (IQR) | 11 (6 – 22) | 10 (6 – 19) | 13 (7 – 30) | 0.014 |

**Supplementary Table 2: Outcomes of opioid naïve population based on admission to critical care**

|  | Overall  (n = 434) | Hospital cohort  (n = 217) | Critical care cohort  (n = 217) | *p*-value |
| --- | --- | --- | --- | --- |
| Prescription count after discharge – median (IQR) | 4 (1 – 10) | 3 (1 – 9) | 4 (0 – 12) | 0.500 |
| Max WHO analgesic ladder after discharge – n (%)  None  Non-opioid (step 1)  Weak opioid (step 2)  Strong opioid (step 3) | 101 (23.3%)  113 (26.0%)  141 (32.5%)  79 (18.2%) | 42 (19.4%)  71 (32.7%)  76 (35.0%)  28 (12.9%) | 59 (27.2%)  42 (19.4%)  65 (30.0%)  51 (23.5%) | <0.001 |
| Chronic opioid prescription after discharge – n (%) | 190 (43.8%) | 96 (44.2%) | 94 (43.3%) | 0.800 |

**Supplementary Figure 2: Maximum WHO analgesic ladder prescription following discharge stratified by age and admission to critical care**

**
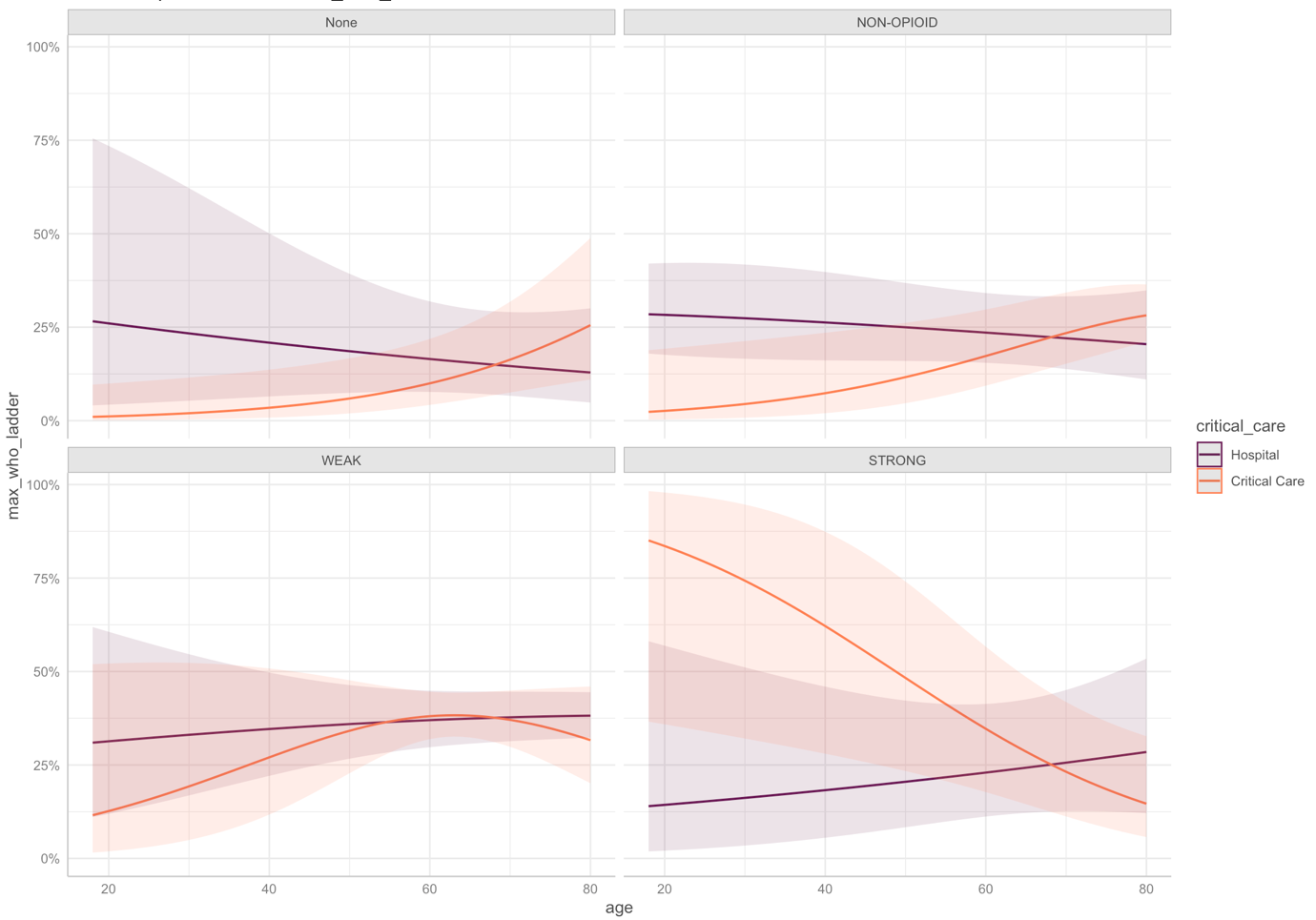
**
